# Ice holes microrefugia harbor genetically and functionally distinct populations of *Vaccinium vitis-idaea* (Ericaceae)

**DOI:** 10.1038/s41598-023-39772-5

**Published:** 2023-08-11

**Authors:** Rita Tonin, Selina Wilhelmi, Mehmet Gültas, Renato Gerdol, Ovidiu Paun, Emiliano Trucchi, Armin Otto Schmitt, Camilla Wellstein

**Affiliations:** 1 Faculty of Agricultural, Environmental and Food Sciences, Free University of Bozen-Bolzano, 39100 Bozen, Italy; 2 Breeding Informatics Group, Department of Animal Sciences, University of Göttingen, 37075 Göttingen, Germany; 3 Center for Integrated Breeding Research (CiBreed), 37075 Göttingen, Germany; 4 Department of Forest Genetics and Forest Tree Breeding, University of Göttingen, 37077 Göttingen, Germany; 5 Faculty of Agriculture, South Westphalia University of Applied Sciences, 59494 Soest, Germany; 6 Department of Environmental and Prevention Sciences, University of Ferrara, 44121 Ferrara, Italy; 7 Department for Botany and Biodiversity Research, University of Vienna, 1030 Vienna, Austria; 8 Department of Life and Environmental Science, Università Politecnica delle Marche, 60131 Ancona, Italy

**Keywords:** Biogeography, Conservation biology, Ecological genetics, Molecular ecology, Ecology, Ecology, Plant genetics, Genetics, Population genetics, Genetic variation

## Abstract

In the mountain terrain, ice holes are little depressions between rock boulders that are characterized by the exit of cold air able to cool down the rock surface even in summer. This cold air creates cold microrefugia in warmer surroundings that preserve plant species probably over thousands of years under extra-zonal climatic conditions. We hypothesized that ice hole populations of the model species *Vaccinium vitis-idaea* (Ericaceae) show genetic differentiation from nearby zonal subalpine populations, and high functional trait distinctiveness, in agreement with genetic patterns. We genotyped almost 30,000 single nucleotide polymorphisms using restriction site-associated DNA sequencing and measured eight functional traits indicative of individual performance and ecological strategies. Genetic results showed high differentiation among the six populations suggesting isolation. On siliceous bedrock, ice hole individuals exhibited higher levels of admixture than those from subalpine populations which could have experienced more bottlenecks during demographic fluctuations related to glacial cycles. Ice hole and subalpine calcareous populations clearly separated from siliceous populations, indicating a possible effect of bedrock in shaping genetic patterns. Trait analysis reflected the bedrock effect on populations’ differentiation. The significant correlation between trait and genetic distances suggests the genetic contribution in shaping intraspecific functional differentiation. In conclusion, extra-zonal populations reveal a prominent genetic and phenotypic differentiation determined by history and ecological contingency. Therefore, microrefugia populations can contribute to the overall variability of the species and lead to intraspecific-driven responses to upcoming environmental changes.

## Introduction

Quaternary climatic fluctuations were responsible for species’ extinctions and range shifts, which strongly influenced the present distribution of plant species^1^. During glacial periods, many boreo-alpine species experienced range retractions and southwards shifts in the face of ice expansion^2^. During interglacial periods, these species recolonized the higher latitudes or elevations as a consequence of rising temperature^3,4^. While plant species thereby mainly tracked suitable environments, another phenomenon of extinction avoidance during both glacial and interglacial periods has been reported: Local spots characterized by stable microclimatic conditions, the so-called microrefugia, enabled long-term persistence of small populations within largely unfavorable geographical regions^5^. In the fast-warming climate of the Anthropocene^6^ the existence of cold microhabitats within a warmer surrounding is even more important for the persistence of boreo-alpine plant species at the retracting margin of their area^7,8^. The persistence of these cold-adapted species over long periods of time within such local spots is expected to result in the genetic and phenotypic differentiation of their isolated populations, making them of particular interest for species conservation^9–11^.

Genetic differentiation can be mirrored at the phenotypic level. However, phenotypic differences among populations can also be the result of phenotypic plasticity, which allows an immediate response of organisms to the environment^12^. Plant functional traits are individual phenotypic characteristics that give information about important plant functions, e.g., photosynthetic capacity, growth, and competitive ability^13^. Given the correlation between functional traits and individual fitness and success, they would easily differ among populations experiencing various environmental conditions.

In the mountain terrain, ice holes represent montane cold inter-glacial refugia^14–16^. They are characterized by depressions between rock boulders, which exhibit cold exiting air able to cool down the rock surface of the holes even during summer. This phenomenon, and the resulting peculiar extra-zonal climate is an exceptional consequence of landslides, which have presumably originated during repeated post-glacial events^17^. It depends on air flows descending from the upper edge of the landslide to the base becoming cooler and denser while passing through the cold humid rock blocks. The air that exits the ice holes accumulates over concave landforms about five meters deep, creating as permanent cold air pool that allows the growth of subalpine and alpine species which are absent in the surrounding area^17–19^. Populations of subalpine and alpine species growing today in ice holes might represent relict populations that have been left behind with the shifts of the species main range toward higher altitudes during the Holocene^20^. The isolation of these small populations for thousands of years may have left signatures in their genome^9^. Indeed, isolation maintained over long periods can lead to genetic differentiation of marginal populations as shown for *Vaccinium vitis-idaea* L. (Ericaceae) in Japan^21^. In line with this, further studies showed that genetic diversity is more strongly preserved in areas where the species persisted for long periods such as glacial refugia^8,22^. On the contrary, genetic diversity is expected to decrease during range expansion such as in case of colonization of formerly glaciated areas, due to repeated bottlenecks^23,24^. Up to our knowledge, our study is the first to investigate this hypothesis for inter-glacial microrefugia in the Alps.

Our study species *Vaccinium vitis-idaea* is one of the most frequent subalpine species, characteristic of the extra-zonal vegetation of ice holes of calcareous as well as siliceous bedrock. This species has been analyzed at the phenotypic and/or genotypic level in different countries by 13 extant studies, however, only two of them linked the genetic and phenotypic diversity focusing on agronomically important traits (Supplement file S1, Table S1). Therefore, knowledge on the relations of genetic and phenotypic functional traits’ diversity in natural environments represent a research gap. Interestingly, another study dealt with in Japanese so-called wind-holes, which are microrefugia similar to ice holes^16^.

**Figure 1:**
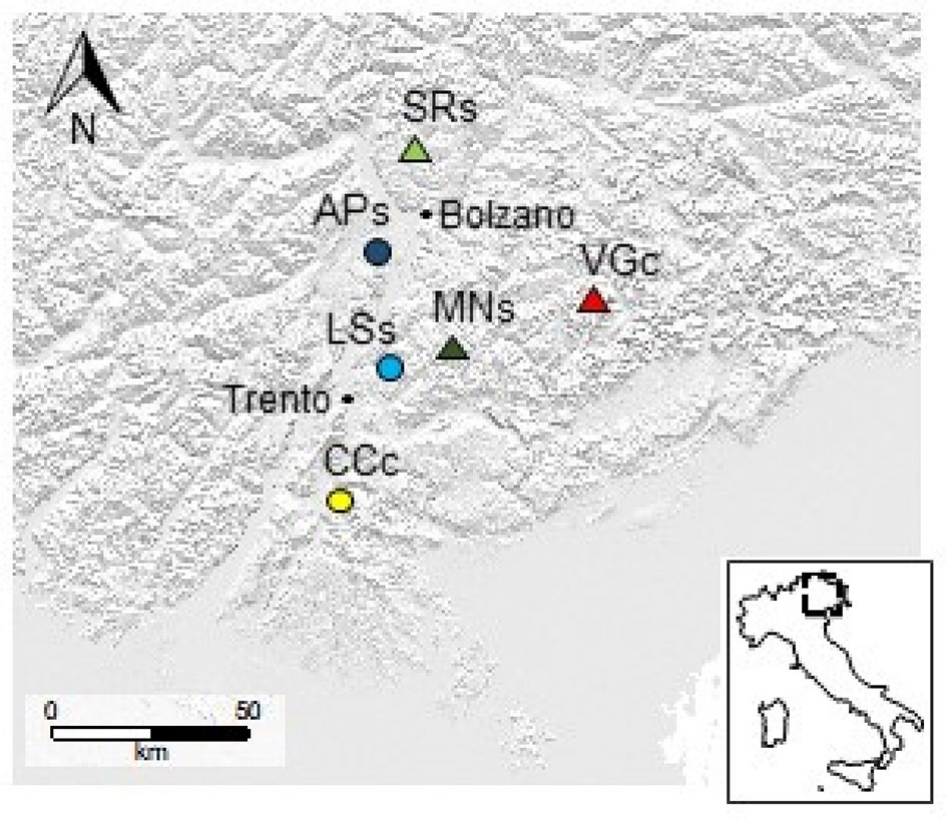
Map of the investigated sites. The circles indicate the ice holes (APs = Appiano, LSs = Lases, CCc = Cornacalda), the triangles indicate the subalpine areas (SRs = Monti Sarentini, MNs = Passo Manghen, VGc = Val di Garés). The letters “s” and “c” following the site name indicate the siliceous or calcareous bedrock, respectively. The grey scale is based on the standard south–east shading applied to visualize the topography in geographical maps. The map was created using the software R (R Core Team, 2019, version 3.6.0, https://www.R-project.org/).

The goal of this study was to investigate the genetic structure and functional variation of ice hole populations of the subalpine species *V. vitis-idaea* and their relationship with populations growing in the current main distributional range of the species, i.e., the subalpine vegetation belt (Fig. 1). Given their isolated occurrence, we asked whether ice hole populations are well differentiated from their subalpine counterparts therefore showing genetic profiles that can shed light on the movement of the species during the last climatic fluctuations enlarging the species genetic pool. In this context we expect that ice-holes harbor a higher genetic diversity compared to the later colonized subalpine areas which might have suffered bottleneck events. In a previous study on a larger pool of species, we found that key leaf functional traits differed between populations of *V. vitis-idaea* growing in ice holes and in the subalpine belt^25^. Hence, we further asked whether any putative genetic differentiation is also reflected at the phenotypic level as differentiated intraspecific patterns of functional trait variation.

## Results

### Demultiplexing, SNP calling and genetic diversity

For the 34 samples, a total of 141.7 million high quality read pairs were retained after demultiplexing and quality check. A total of 29,148 loci with a maximum of ten polymorphic nucleotide positions were retained after de novo catalog building, SNP calling and filtering. For each population, we recorded the number of private alleles and nucleotide diversity (π) (Table 1). Nucleotide diversity was lowest at the two subalpine populations growing on silicates (i.e., SRs and MNs). The negative inbreeding coefficient (F_IS_) shown by all populations indicates an excess of heterozygotes (Table 1). Gene flow estimates (Nm) were always lower than one, suggesting a marginal role of gene flow compared to drift and/or local selection (Table 2). The highest Nm values were found between APs and LSs and between LSs and CCc, i.e., between geographically close ice holes. The lowest Nm value was shown for the pair MNs and SRs, followed by SRs and VGc, i.e., between subalpine habitats.

**Table 1:** Number of private alleles, nucleotide diversity (π) and inbreeding coefficient (*F*_IS_) for each *V. vitis-idaea* population investigated here (APs = Appiano, LSs = Lases, CCc = Cornacalda, SRs = Monti Sarentini, MNs = Passo Manghen, VGc = Val di Garés).

Habitat	Bedrock	Population	Private alleles	π	F_IS_
Ice hole	Silicate	APs	2295	0.00022	− 0.07
Ice hole	Silicate	LSs	2912	0.00024	− 0.07
Ice hole	Carbonate	CCc	2090	0.00022	− 0.09
Subalpine	Silicate	SRs	1488	0.00017	− 0.11
Subalpine	Silicate	MNs	2451	0.0002	− 0.12
Subalpine	Carbonate	VGc	2630	0.00024	− 0.06

**Table 2:** Number of migrants (Nm) inferred based on private alleles.

Pop	APs	LSs	CCc	MNs	SRs	VGs
APs		0.64	0.45	0.35	0.41	0.41
LSs			0.54	0.45	0.52	0.5
CCc				0.3	0.34	0.38
MNs					0.25	0.32
SRs						0.28
VGc						

Pairwise comparisons of the six populations of *V. vitis-idaea* (APs = Appiano, LSs = Lases, CCc = Cornacalda, SRs = Monti Sarentini, MNs = Passo Manghen, VGc = Val di Garés) are based on 29,148 SNPs.

### Genetic structure

**Figure 2:**
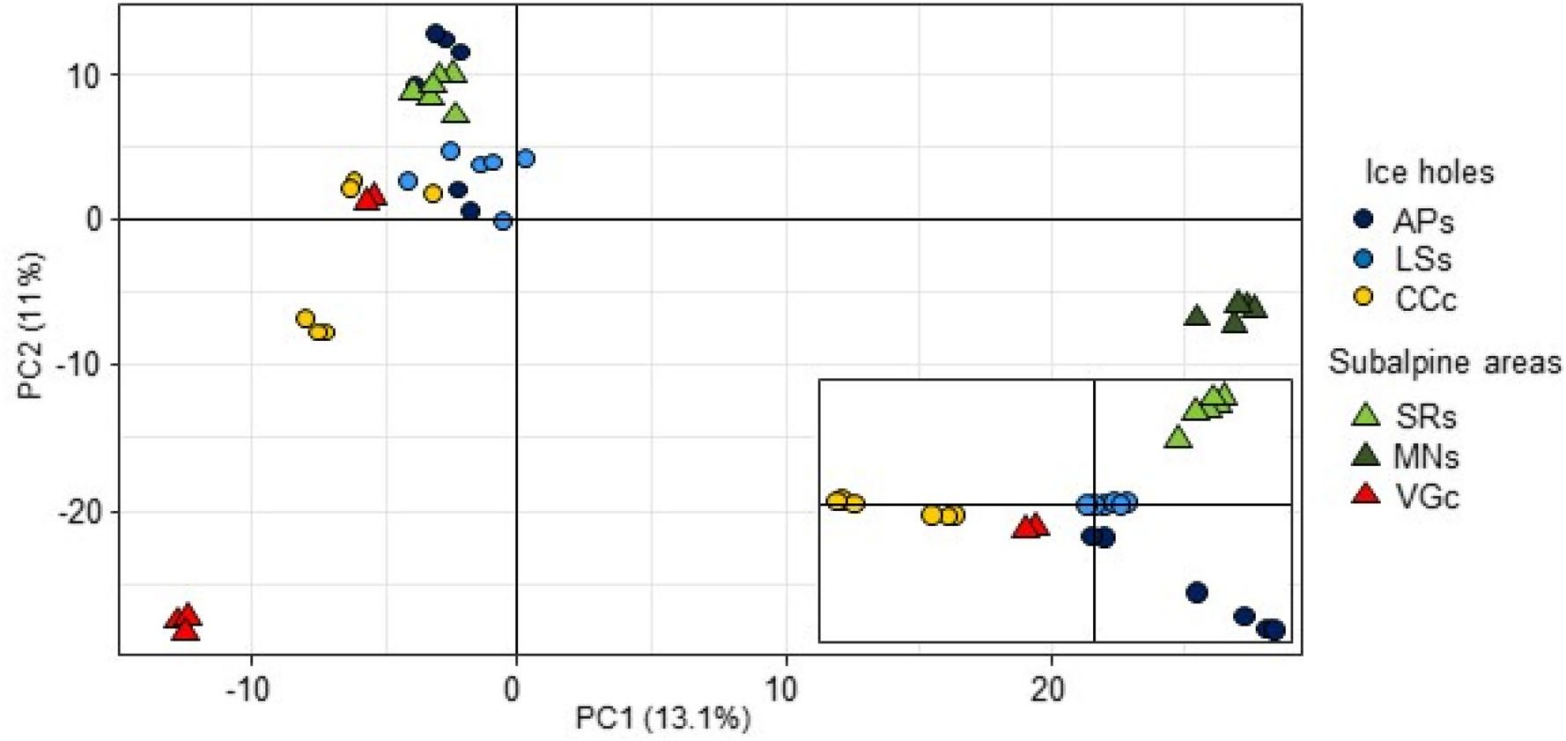
Principal component analysis of the 34 individuals of *V. vitis-idaea* based on the genetic data. The symbols represent the origin of the individuals according to the legend. Inset: PCA on the central group: For a detailed observation of the central main group identified by the PCA, the analysis was repeated excluding MNs population and the three individuals of the VGc populations that fall outside the central main group. The first PCA axis explains 12.9% and the second axis 11.8% of the total variation.

Interpopulation pairwise *F*_ST_ values were on average higher between subalpine populations than between ice hole populations (Table 3). The highest *F*_ST_ values were those obtained by the comparison of MNs population with the others, while the lowest values were those detected for comparisons involving LSs (Table 3). The first two PCA axes based on 29,148 SNPs explained 24.1% of the variance (Fig. 2). They clearly separated the subalpine populations MNs from ice holes populations that clustered together at the center of the plot (APs, LSs and CCc) also with the subalpine SRs population. Three individuals of the subalpine VGc population were also separated from ice holes while two VGc individuals were located close to the ice hole populations, especially to CCc. To observe in detail the central main group identified by the PCA, we repeated the analysis excluding MNs population and the three individuals of the VGc populations that fall outside the central main group (Fig. 2). In this case, the first two axes of the PCA explained 24.7% (PC1 12.9% and PC2 11.8%) of the variance. The ice hole population LSs is positioned at the center of the plot, from where ice holes APs and CCc as well as subalpine SRs populations split off. The two subalpine VGc individuals clustered between LSs and CCc.

**Table 3:** F_ST_ values calculated for each pairwise comparison of the six populations of *V. vitis-idaea* (APs = Appiano, LSs = Lases, CCc = Cornacalda, SRs = Monti Sarentini, MNs = Passo Manghen, VGc = Val di Garés).

	APs	LSs	CCc	SRs	MNs	VGc
APs		0.119	0.161	0.171	0.263	0.172
LSs			0.138	0.146	0.233	0.147
CCc				0.188	0.277	0.164
SRs					0.298	0.202
MNs						0.288
VGc

An additionally performed clustered co-ancestry heatmap drawn with fineRADstructure (Fig.S2) clearly clustered the populations by bedrock, with CCc and VGc populations appearing rather intermixed and forming a group separated from siliceous populations. Subalpine siliceous populations MNs and SRs appeared to be quite homogeneous. Ice holes population APs appeared intermixed with LSs.

**Figure 3:**
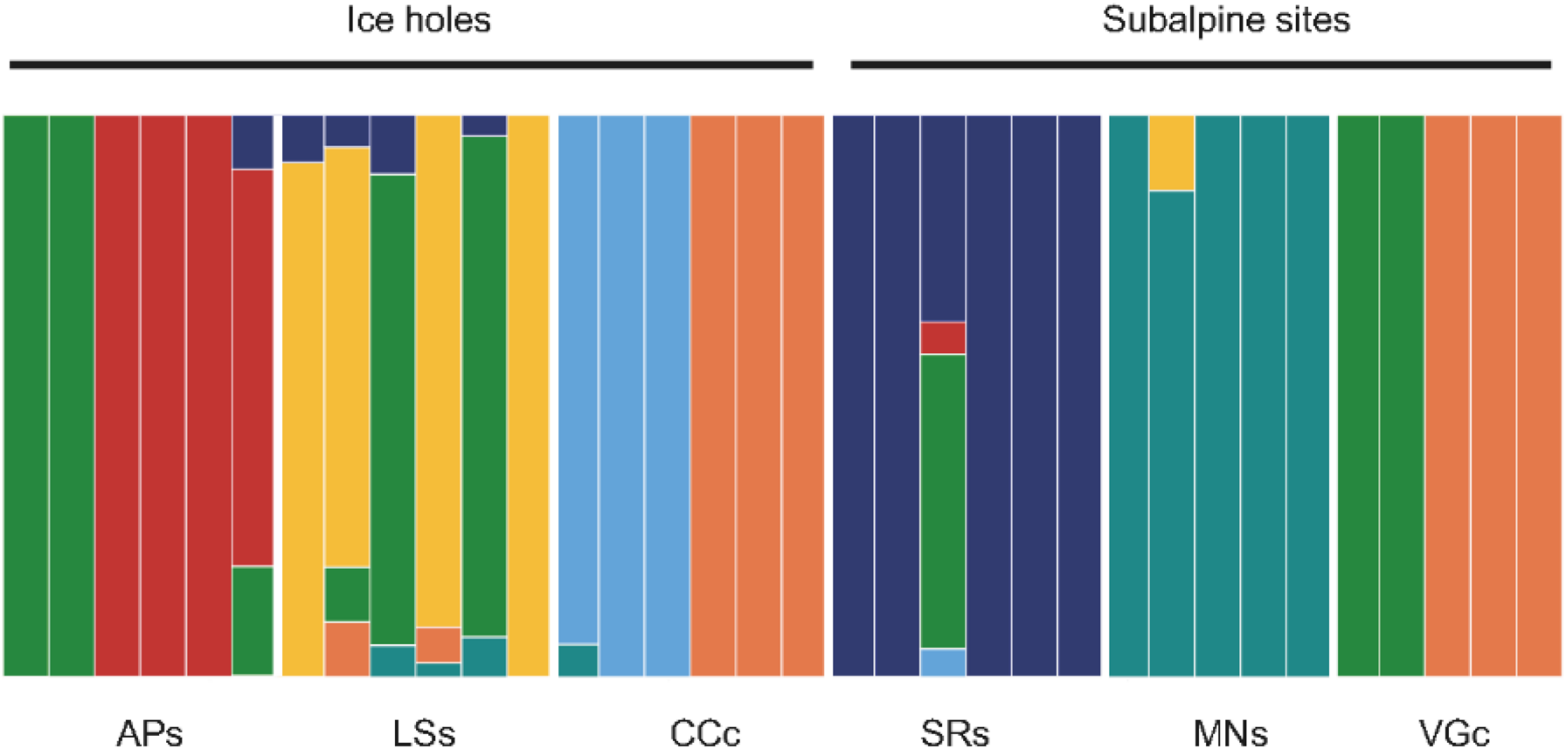
Proportional assignment of the individuals of *V. vitis-idaea* from the three ice holes (APs; LSs; CCc) and the three subalpine sites (MNs; SRs; VGc) to the seven groups identified by Structure analysis. Each of the seven groups is displayed by a different color.

Structure analysis revealed seven main genetic clusters (K = 7). Subalpine siliceous populations MNs and SRs appeared well separated from the others being characterized by one prevalent structure cluster (Fig. 3). Three individuals of the calcareous subalpine population VGc and three individuals of the calcareous ice holes population CCc shared the same structure cluster (Fig. 3). Ice hole populations APs and LSs shared a common genetic cluster.

When habitat was considered as the highest grouping factor, AMOVAs recorded most of the variance within populations, i.e., 88.14%, whereas the habitat classification did not contribute to explain any of the genetic variance (Table 4). Similar results have been obtained considering bedrock as the highest grouping level, although the bedrock classification explained only 0.9% of the variance. Negative variance components, such as the one among groups of habitats in our analysis, can sometimes occur, because they are rather covariances. Most likely, these negative variance components indicate an absence of genetic structure.

**Table 4:** Results of AMOVAs of *V. vitis-idaea* with habitat (ice holes and subalpine areas) and bedrock (siliceous and calcareous) as higher-level groups.

Source of variation	d.f	Sum of squares	Variance components	Percentage of variation	Fixation index
Habitat					
Among groups	1	4030.87	− 5.68	− 0.3	*F*_CT_ = 0.00
Among populations within groups	4	16870.22	227.26	12.16	*F*_SC_ = 0.12***
Within populations	62	102,096.7	1646.72	88.14	*F*_ST_ = 0.12***
Bedrock					
Among groups	1	4585.30	17.67	0.94	*F*_CT_ = 0.01*
Among populations within groups	4	16,315.79	214.56	11.42	*F*_SC_ = 0.11***
Within populations	62	102,096.65	1646.72	87.64	*F*_ST_ = 0.12***

Fixation indices (*F*_CT_ = fixation index among groups; *F*_SC_ = fixation index among populations within groups; *F*_ST_ = fixation index within populations) and levels of significance ((*) *P* < 0.1; (***) *P* < 0.001) are reported. The analysis is based on the six populations (APs = Appiano, LSs = Lases, CCc = Cornacalda, SRs = Monti Sarentini, MNs = Passo Manghen, VGc = Val di Garés).

Results of the Mantel–Haenszel test for the statistical correlation between geographic and genetic distance matrices indicated significant IBD between populations. The correlation coefficient was 0.46 with a *p*-value of 10^–5^.

### Functional traits and genetics correlation

**Figure 4:**
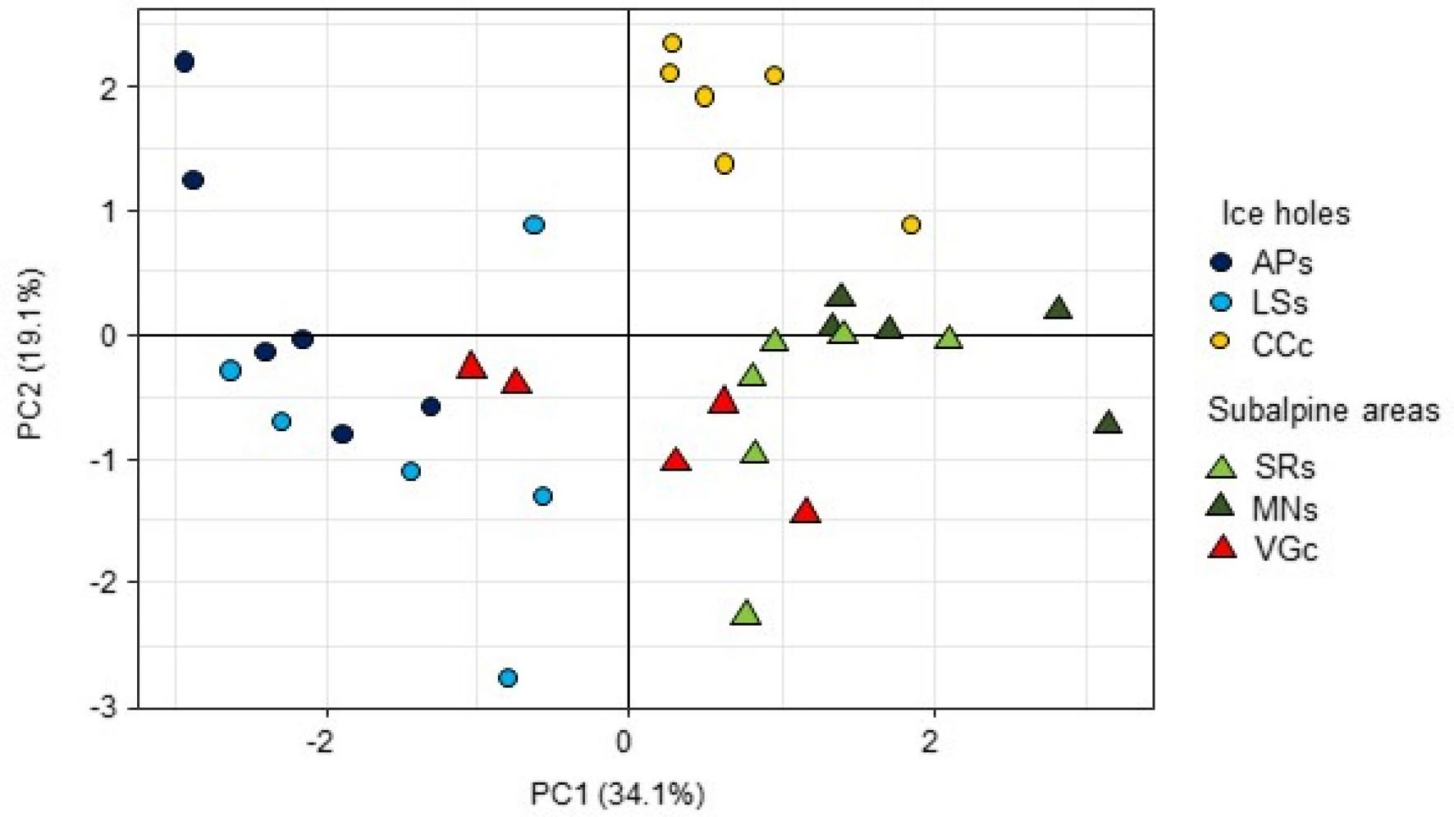
Principal component analysis of functional traits (leaf area, specific leaf area, leaf dry matter content, leaf nitrogen content, leaf phosphorous content, N:P ratio, stomatal density and stomatal length) of individuals of *V. vitis-idaea*.

The first two axes of the PCA on functional trait values explained 53.13% of the traits’ variance (Fig. 4).

**Figure 5:**
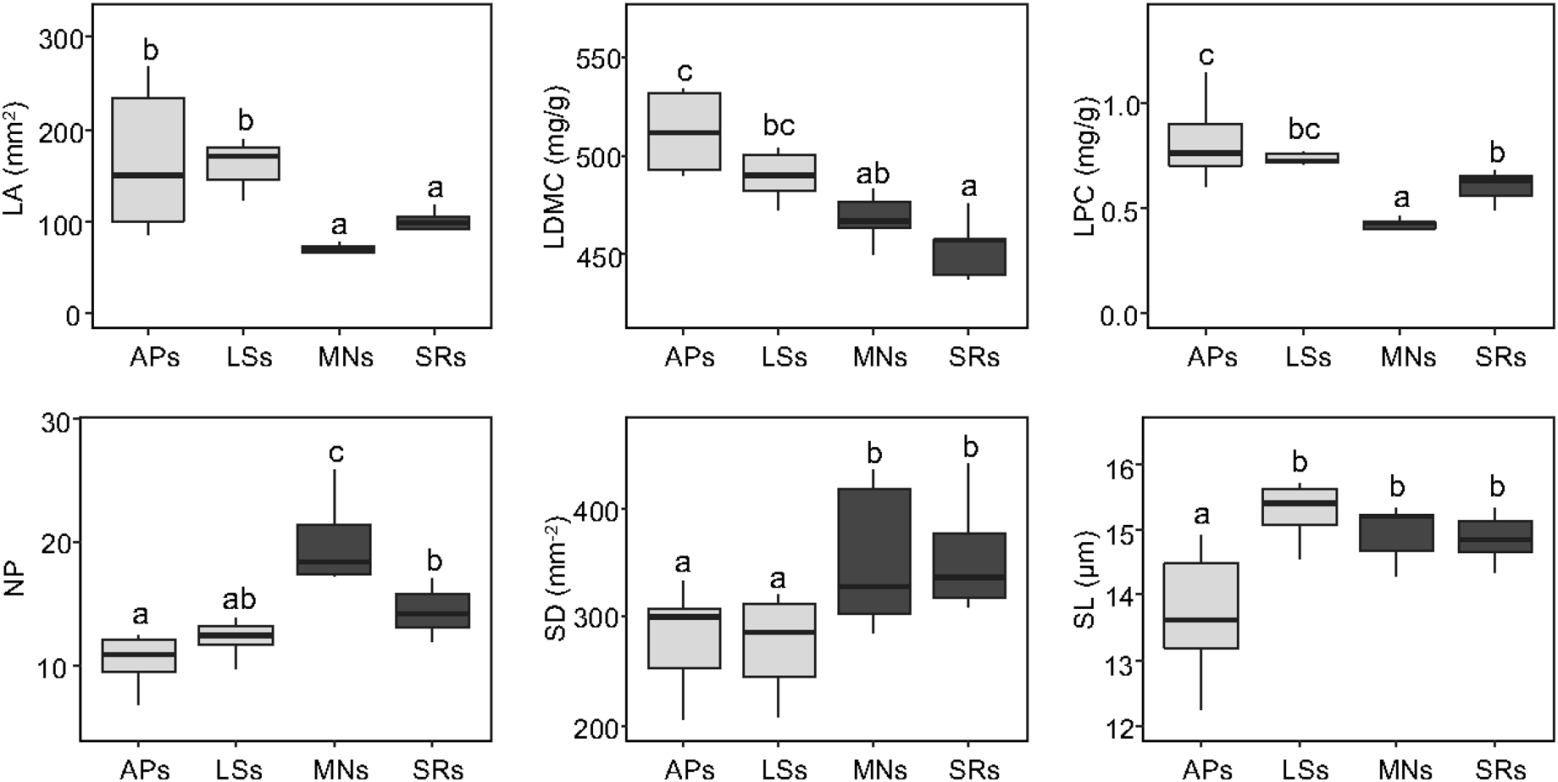
Box-and-whisker plots for six functional traits of *V. vitis-idaea* showing significant differences between individuals growing in siliceous ice holes (APs; LSs) and siliceous subalpine areas (MNs; SRs) according to ANOVA. LA, leaf area; LDMC, leaf dry matter content; LPC leaf phosphorous content; NP, N:P ratio; SD, stomatal density; SL, stomatal length. Different letters indicate significant differences (*P* < 0.05) between populations according to ANOVA.

**Figure 6:**
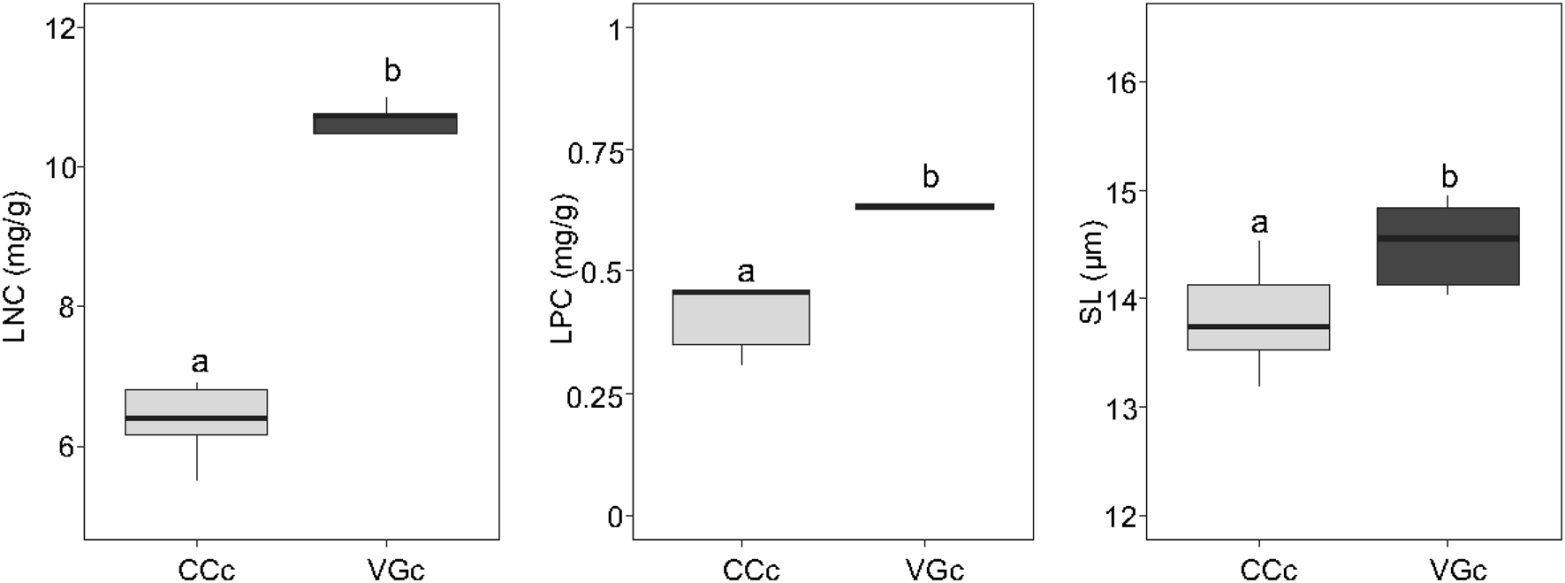
Box-and-whisker plots for three functional traits of *V*. *vitis-idaea* showing significant differences between individuals growing in the calcareous ice hole of Cornacalda) (CCc) and the calcareous subalpine area Val di Garés on calcareous substrate (VGc) according to ANOVA. LNC, leaf nitrogen content; LPC, leaf phosphorous content; SL, stomatal length. Different letters indicate significant differences (*P* < 0.05) between populations.

The samples from siliceous and calcareous subalpine habitats were grouped in the right and in the middle lower part of the diagram. The ice hole samples were sharply separated in the PCA diagram in relation to bedrock (Fig. 4). The ANOVAs on functional traits showed significant effects of habitat (ice hole vs subalpine) and habitat × bedrock interaction for LA, LDMC, LNC, LPC, NP, and SD. Stomatal length was the only trait showing a significant effect of bedrock while SLA was unaffected by any of the two factors (Table 5). In particular, the ice hole populations on siliceous bedrock clearly exhibited higher LA and lower SD, and a tendency toward higher LDMC and LPC, and lower N:P compared to the subalpine populations (Fig. 5). However, stomatal length was different between individuals sampled in the LS ice hole exhibiting higher values than individuals of the AP ice hole (Fig. 5). On calcareous bedrock, ice hole individuals exhibited significantly lower values of LNC, LPC and SL than their subalpine counterparts (Fig. 6).

**Table 5:** Results of ANOVAs for each single investigated functional trait in individuals of *V. vitis-idaea*.

Trait	Variables	F value	*p*-value
LA	**Habitat**	**19.29**	**0.00**
	Bedrock	0.14	0.71
	Habitat*bedrock	**11.90**	**0.00**
SLA	Habitat	1.89	0.18
	Bedrock	0.78	0.39
	Habitat*bedrock	0.77	0.39
LDMC	**Habitat**	**20.05**	**0.00**
	Bedrock	0.26	0.62
	Habitat*bedrock	**8.07**	**0.01**
LNC	**Habitat**	**8.11**	**0.01**
	Bedrock	0.14	0.71
	Habitat*bedrock	**16.04**	**0.00**
LPC	**Habitat**	**4.42**	**0.04**
	**Bedrock**	**9.46**	**0.00**
	Habitat*bedrock	**28.60**	**0.00**
NP	**Habitat**	**11.00**	**0.00**
	**Bedrock**	**5.38**	**0.03**
	Habitat*bedrock	**6.80**	**0.01**
SD	**Habitat**	**5.59**	**0.02**
	Bedrock	0.02	0.65
	Habitat*bedrock	**6.43**	**0.02**
SL	Habitat	2.61	0.12
	**Bedrock**	**4.22**	**0.05**
	Habitat*bedrock	0.08	0.79

Each functional trait was analyzed as the response variable, habitat, bedrock and their interaction as explanatory variables. Significant results (*P* < 0.05) are reported in bold.

The result of the Mantel–Haenszel test assessing statistical correlation between phenotypic and genetic distance matrices was significant at the level of alpha = 0.05, with a correlation coefficient of 0.18 and a simulated *p*-value of 10^–4^. This meant that at least a part of the phenotypic variability can be explained by genotypic variability.

## Discussion

Our results revealed a certain degree of differentiation among the six *V. vitis-idaea* populations, as shown for example by the increased values of the pairwise *F*_ST_ and the significant IBD. The relatively high number of private alleles pointed towards a long-term isolation of the populations^26^. As a consequence, the number of migrants calculated on private alleles confirmed a marginal contribution of gene flow in shaping genetic composition in the six populations, suggesting a more important role of drift and/or local selection in shaping genetic patterns.

Given their extra-zonal location, the small *V. vitis-idaea* populations persisting in microrefugia presumably survived isolation thanks to clonal reproduction and the evolution of mechanisms keeping inbreeding depression low^10,27^. Low levels of inbreeding depression are expected to be related to high rates of self-fertilization considering that inbreeding should allow expression and purge of deleterious alleles. However, in our case, the negative values of F_IS_ for ice hole populations indicate an excess of heterozygotes, which suggests the existence of some mechanisms avoiding selfing. This contradiction can be explained by the capacity of *V. vitis-idaea* to reproduce both clonally and sexually. Indeed, it has been mathematically demonstrated through simulations that *F*_IS_ can be maintained at high levels in partially clonal populations^28^. For this reason, this type of population perpetuated traces of their past for a longer period than their counterparts that reproduce exclusively sexually^28^. Furthermore, asexual reproduction is expected to conserve heterozygosity through generations by limiting the segregation of alleles^29^. Individuals of *V. vitis-idaea* can persist for hundreds of years^16,30^ by clonal reproduction. In addition to these mechanisms^31^, showed that individuals in ice holes in Japan were tetraploids, which may contribute to high genetic diversity and heterozygosity. Thus, it is not surprising to find that the individuals from different ice hole populations are intermixed, as shown by results of the fineRADstructure and Structure analyses. Indeed, individuals from the siliceous ice holes APs and LSs appear to form a cluster separated from the subalpine siliceous populations, especially MNs, as shown also by PCA results. The fact that APs and LSs did not clearly separate into defined populations indicated the maintenance of genetic variability of the ice holes individuals.

Previous studies showed that genetic diversity for many species is expected to decrease during range shift expansion while it is more strongly preserved in areas where the species persisted for long periods such as glacial refugia^8,22^. This is because colonization of formerly glaciated areas involved repeated bottlenecks^23,24^. Hence, ice hole stands are probably older than the subalpine SRs and MNs populations that might have experienced one or more bottleneck events. This supports the idea that these genetic groups may stem from a larger genetic pool and are now still in part preserved in microrefugia populations. This hypothesis is reinforced by *F*_ST_ results, showing higher values between subalpine populations than between ice holes populations.

Calcareous populations, i.e., ice hole CCc and subalpine VGc, appear to be more related to each other than to populations belonging to their own habitat. The choice of considering both bedrock types in our study was made to include as much variability as possible. This is important since these two bedrock types are widespread in the alpine landscape^32^. As to bedrock differences, the AMOVA results showed that diverse bedrocks explained a slightly significant part of genetic variation between populations. The clustered fineRADstructure coancestry heatmap clearly separated calcareous populations from the siliceous ones. These results are supported also by Structure results, that showed a common genetic cluster shared by some CCc and VGc individuals and the ordination plot that shows a gradient of CCc and VGc individuals departing from the central group towards the lower part of the left quadrant. This affinity between the two populations on calcareous bedrock may indicate a certain relevance of bedrock in shaping genetic patterns or in selecting genotypes from founder populations in our case study^33^.

Stronger differences between populations growing on diverse bedrock types were found at the phenotypic level in our study. Indeed, the ordination plot based on functional traits confirmed a phenotypic differentiation depending on habitat and bedrock. Furthermore, from our results on functional variability for each investigated trait, it was possible to distinguish two different strategies adopted by ice hole individuals according to the bedrock differentiation. On siliceous bedrock, *V. vitis-idaea* individuals modified their traits towards a more conservative strategy in ice holes, i.e., exhibiting lower stomatal density, and higher LA and LDMC. These results are in line with the findings reporting functional differentiation of several plant species in siliceous ice holes microrefugia compared to subalpine sites^25^. The fact that high LDMC values are inversely related to relative growth rate^34,35^ indicates that ice hole individuals follow a more conservative strategy, with low growth rates and producing more resistant and longer-lived leaves compared to subalpine individuals^36^. This may confirm the adoption of an ecological strategy in ice holes related to the survival of individuals for a long time investing mainly in the capacity to conserve resources and repair cellular components decreasing leaf turnover^37,38^. Increased LA would explain lower SD values in ice hole populations than subalpine ones. Indeed, stomatal number per mm^2^ is inversely proportional to LA^39,40^. Higher LPC in ice holes could depend on the location of the siliceous sites in the elevational zone of montane forests, known to be rich in soil organic matter^32^. On calcareous bedrock, the lower amount of nutrient content in microrefugia is probably due to environmental conditions experienced by *V. vitis-idaea* in the ice hole^25^. Indeed, in the CCc ice hole, plants are growing directly between rocks, where soil is almost absent^17^. Shorter SL can instead depend on the tendency of the species to adjust stomatal size instead of stomatal density under different environmental conditions^41^.

Interestingly, we found a significant correlation between functional traits and genetic distances between the six populations. This may indicate that functional trait variation is not only the result of phenotypic modifications induced by environment and climate, but that long-term genetic variation accounted for intraspecific functional variability as well^42^. In spite of their small size, microrefugia populations can therefore contribute significantly to the overall diversity of this species while providing information about the history of the species itself. Furthermore, the bedrock effect identified at both genetic and phenotypical level indicates a certain relevance of ecological conditions in shaping genetic composition and distribution of different populations of the same species in our case study. Interestingly, our results are also in line with the finding from^31^ on the same species in Japan that populations at lower elevations contained unique ecotypes suited to persistence in isolated situations. Understanding how these small populations persisted in isolation outside the main distributional range of the species could provide useful information on future species’ reaction to ongoing climatic modifications^8^. Therefore, studies of interglacial microrefugia and of the species hosted within are of high interest for conservation purposes. Regarding data limitation, we acknowledge that our results on the difference of genetic structures and phenotypic traits among habitat or bedrock types refer to the studied populations of this case study and the restricted sample size prevents from further generalization. Our study includes all ice hole microsites that are existing in the region of South-Tyrol and Trentino region at the southern side of the Alps. However, we encourage further studies in other areas of the Alps as more sites would be needed to support our findings and generalize them for the alpine environment.

## Material and methods

### Sites selection and sample collection

The sampled species, i.e., *Vaccinium vitis-idaea* L*.* (lingonberry), is not protected according to IUCN standards. The plant collection and use was in accordance with all the relevant guidelines. *V. vitis-idaea* leaf samples were collected at six localities, in three ice holes and in three subalpine sites located in the south-eastern Italian Alps (Fig. 1). The ice holes were identified based upon the extant literature^17–19,43–45^. Two ice holes, Appiano (Aps, 46°26′N 11°14′E, 530 m a.s.l., Province of Bolzano) and Lases (LSs, 46°8′N 11°13′E, 660 m a.s.l., Province of Trento), lie on siliceous bedrock while the third, Cornacalda (CCc, 45°51′N 11°3′E, 700 m a.s.l., Province of Trento), lies on calcareous bedrock. To make the bedrock of each locality clear, a letter indicating its siliceous (s) or calcareous (c) nature was added to the site acronym. The three ice holes, although being all located in large block fields, differ rather strongly from each other in terms of topography. In particular, the calcareous site differs substantially from the two siliceous sites (Fig. S1). The calcareous ice hole is more detrital, with less soil accumulating among the blocks (Fig. S1a). The siliceous ice holes are more similar to each other despite topographical differences. The APs ice hole is located in a small circular depression surrounded by broadleaf deciduous woodlands (Fig. S1b). Likewise, the LSs ice hole is surrounded by broadleaf deciduous woodlands but has a more elongated shape (Fig. S1c).

The subalpine sites are located at about 2000 m a.s.l.. As the ice hole sites, the subalpine sites are on either siliceous or calcareous bedrock. The siliceous bedrock sites are located nearby Passo Manghen, on the Lagorai chain (MNs, 46° 10' N 11° 26' E, 2060 m a.s.l., Province of Trento), and on Monti Sarentini (SRs, 46° 38' N 11° 18' E, 1917 m a.s.l., Province of Bolzano). The site on calcareous bedrock is located in Val di Garés (VGc, 46° 17' N 11° 52' E, 2015 m a.s.l., Province of Belluno) (Fig. 1). For the ice hole sites, the mean annual temperature (MAT) is 13 °C in APs (data for Pianizza di Sopra—Caldaro, 495 m a.s.l., http://meteo.provincia.bz.it/), 11 °C at LSs (data for Lases, 700 m a.s.l., https://www.meteotrentino.it/) and 10 °C at CCc (data for Terragnolo, 800 m a.s.l., https://www.meteotrentino.it/). The MAT for the subalpine sites is 3 °C at MNs (data for Passo Manghen climatic station, 2035 m a.s.l., https://www.meteotrentino.it/), 1 °C at SRs (data for Corno del Renon, 2260 m a.s.l., http://meteo.provincia.bz.it/) and 4 °C at VGc (according to WorldClim datasets at a spatial resolution of 30 arc second ^46^). The mean annual precipitation is 815 mm at APs, 1043 mm at LSs, 822 mm at CCc, 1150 mm at MNs, 800 mm at SRs and 1450 mm at VGc.

Sampling was carried out at the peak of the growing season 2016, namely in July in the ice holes and in August in the subalpine sites. We sampled six healthy, adult individuals because of the small area of ice holes (ca. 20 m^2^). Sampled individuals were distant within each locality, as the species *V. vitis-idaea* can extend by clonal growth for more than 20 cm per year through horizontal stems^47^. For MNs and VGc, individuals were subsequently reduced from six to five, owing to technical problems in DNA extraction. For each individual, we harvested 20 fully expanded leaves: Ten of these were immediately used for functional trait measurements while the other ten were stored in silica gel for the genetic analyses.

### RADseq libraries preparation and sequencing

Genome size was estimated by flow cytometry. DNA was extracted from 50 mg of dry material from each individual plant using the DNeasy Plant Mini Kit (QIAGEN, Hilden, Germany) with addition of up to 3.1% polyvinylpyrrolidone (PVP) to the AP1 lysis buffer to enhance the final yield. The samples were incubated overnight at 65 °C with the mixed buffer and RNase A^48^. RAD libraries were prepared using the *Pst*I restriction enzyme (New England Biolabs, NEB, Ipswich, MA, USA) adapting a protocol that was described in previous studies^49,50^. Starting from a concentration of 200 ng of DNA, each individual sample was digested at 37 °C for 2 h and then heated at 80 °C for 20 min for enzyme inactivation. Eight different P1 adapters were ligated to the digested DNA, pooling together individuals with different adapters to form five sub-libraries. Samples in each sub-library were sheared by sonication using a Bioruptor Pico (Diagenode, Seraing, Belgium) to an average size of ca. 400 bp with three cycles of 30 s “on” and 60 s “off” at 4 °C and then purified with the MinElute Reaction Cleanup Kit (Qiagen). After double-size selection 0.55 × right and 0.7 × left with the SPRIselect Reagent Kit (Beckman Coulter, USA), five different P2 adapters with index barcodes were ligated, one per sub-library. At this point, all samples were pooled in a unique library, in a way that they could be distinguished by different P1 and P2 combinations. Before and after PCR amplification with the Phusion Master Mix (NEB), cleaning and size selection only on the left side (0.75 × before and 0.7 × after PCR) were performed again. The final library was sequenced as 125 bp paired-end reads on an Illumina HiSeq at Vienna BioCenter Cora Facilities (VBCF, http://www.vbcf.ac.at/facilities/next-generation-sequencing/).

### Genotyping and SNP filtering

The raw reads were demultiplexed based on index barcoded P2 adapters using the Picard BamIndexDecoder tool, which is part of the Picard Illumina2Bam package (available from https://github.com/wtsi-npg/illumina2bam). Quality filtering and demultiplexing based on inline barcodes was performed with *process_radtags.pl* available in the STACKS 2.0 Beta 9 package^51^ specifying *Pst*I as the restriction enzyme, rescuing RADtags and barcodes and rejecting low quality reads. For detecting the latter, in a very simplified way, the program uses a sliding window approach to obtain the average quality of a read and its probability of being corrected. If the quality drops below a certain threshold (i.e., a raw phred score of 10), the read is discarded as low quality read. More details on the filtering process are explained in the supplement file S2. These obtained sequence data have been submitted to the NCBI Sequence Read Archive (SRA) database under accession ID PRJNA764567. The resulting reads were processed with *ustacks*, *cstacks* and *sstacks* using a minimum of five reads to call a stack (m = 5), allowing for two mismatches between four stacks to assemble a locus in each individual (M = 4) and one mismatch between loci to build the catalog (n = 1). Paired-end information was introduced during the *tsv2bam* step and processed with *gstacks*, calling variant sites in populations and genotypes in every individual. The catalog was filtered blacklisting loci carrying more than ten SNPs to avoid retention of any merged paralogs using a custom bash script. Using the *populations* program from STACKS 2.0, we selected loci shared by at least 75% of the individuals (r = 0.75) and present in all the populations (*p* = 6). As a first step, only one SNP per locus was called to minimize linkage and the options genepop, vcf and structure were selected to produce the respective files for the following data analyses. As a last step, *population* was run again keeping unchanged the above-mentioned parameters but calling all SNP per locus selecting the RADpainter option in preparation for fineRADstructure^52^. The STACKS code used for the analyses is reported in the supplement file S2.

### Functional traits data

Eight leaf functional traits, namely leaf area (LA), specific leaf area (SLA), leaf dry matter content (LDMC), leaf nitrogen content (LNC), leaf phosphorous content (LPC), N:P ratio (N:P), stomatal density (SD) and stomatal length (SL) were measured. LA, SLA, LDMC and the stomatal traits were measured for five of the ten harvested leaves per individual. The other five leaves were used for nutrient analyses. For each of these traits, individual values were obtained averaging the values of the five leaves measured per individual. To measure LA, SLA and LDMC we determined the fresh weight and surface area of each of these five leaves (CanoScan LiDE 120, Canon). The same leaves were then dried at 70 °C for 72 h, weighed again and used for stomatal analyses^53^. Stomatal impressions were obtained by the clear nail polish method^54^. Two epidermal impressions of the abaxial surface per leaf were analyzed using a light microscope (Leica DMLS, Leica Biosystems, Nußloch, Germany) connected to a digital camera (DeltaPix, Måløv, Denmark) at 400 × magnification. SD (number of stomata × mm^−2^) and SL (guard cell length, µm)^55^ were estimated for five fields of view per impression, i.e., ten per leaf, using the software DeltaPix, v.3.2.× (DeltaPix, Måløv, Denmark). Nutrient traits, i.e., LNC and LPC, were measured from the remaining five leaves per individual by the salicylate method and molybdenum blue method through a continuous flow autoanalyzer (FlowSys; Systea, Anagni, Italy). See Table S1 in the supplement file S1 for more information about the traits and their functional significance.

### Data analyses

The population genetics were analyzed by several different methods described as follows. The genepop file produced with the *populations* program was used to perform a Principal Component Analysis (PCA) in R with the *adegenet* package v. 2.1.1^56^. With the *adegenet* package the file was also adapted to obtain the number of migrants (Nm) based on private alleles through the pairwise comparison of populations in R with the *genepop* package^57^. For each pair of populations, pairwise F_ST_ was calculated with the software VCFtools^58^ using the vcf file obtained by *populations* program. To deeply investigate the structure and the relationships of the populations inferring their recent shared ancestry, both fineRADstructure and Structure analyses were run. The RADpainter file produced from *populations* was used as input file for the fineRADstructure package that was run with standard options and the structure file produced from *populations* was used as input file for the software Structure^59^ (v. 2.3.4) that was employed using the admixture model with correlated frequencies between populations. For each K (from 1 to 12) 10 replicates, 100,000 burn-ins and 200,000 iterations were computed. The K value that fitted the data the best was inferred through Structure Harvester v. 0.6.94^60^. In R, *pophelper* package ^61^ was used to define the admixture proportion resulting from the 10 simulations at the best K and to represent the results through barplots. After conversion of the structure file from *populations* with PGDSpider v. 2.1.1.3^62^, we applied ARLEQUIN v. 3.5.2.2^63^ to assess the genetic structure of the populations. It allowed the calculation of AMOVAs to estimate genetic diversity of populations and higher-level groups, i.e., habitat and bedrock. When habitat was considered as the higher-level group, the three ice hole populations, i.e., APs, LSs, and CCc were distinguished from the three subalpine populations, i.e., SRs, MNs and VGc. In contrast, when bedrock was the higher-level group the four populations occurring on the siliceous bedrock, i.e., APs, LSs, SRs and MNs were distinguished by the two occurring on calcareous bedrock, i.e., CCc and VGc.

Finally, the effect of isolation by distance (IBD) on the genetic structure was investigated through the comparison of geographical and genetic matrices. The genetic matrix was obtained by using the R packages *adegenet* and *poppr*^64^. Geographic distances were calculated as straight-lines including elevational differences. The geographic distance matrix was then tested for statistical correlation with the genetic distance matrix in R through a Mantel–Haenszel test with 100,000 permutations in the *ade4* package v. 1.7-10^65^.

The functional traits’ analysis is done in two steps. First, the analysis of phenotypic traits’ differences, and second, the correlation between the functional traits’ differences and genetic difference. To detect phenotypic differences of individuals between populations of *V. vitis-idaea* growing in the two different habitats, a PCA was performed on functional trait data of LA, SLA, LDMC, LNC, LPC, N:P, SD and SL in R (R Development Core Team 2014, version 3.4.3). Furthermore, to make the intraspecific variability of each trait evident, univariate two-way factorial ANOVAs were performed with each functional trait as dependent variable and habitat, bedrock, and their interaction as independent variable. Significance of differences of means among sites were assessed by Fisher’s LSD post-hoc test. To correlate the functional with the genetic distance, a multidimensional scaling analysis was performed on the distance matrix of the abovementioned traits through the R package *bios2mds*^66^. The Mantel–Haenszel test with 100,000 permutations was then applied to assess the statistical correlation between the genetic and the phenotypic distance matrices. For a conceptual model of relationships among intraspecific functional trait variation, abiotic heterogeneity and genetic diversity we refer to^67^ and the conceptual figure therein.

### Supplementary Information

Supplementary Information 1.

Supplementary Information 2.

## Data Availability

The sequence data is available from the NCBI SRA under the BioProject ID PRJNA764567 (https://dataview.ncbi.nlm.nih.gov/object/PRJNA764567).
