# Supplementary material for: Ice holes microrefugia harbor genetically and functionally distinct populations of Vaccinium vitis-idaea (Ericaceae)
Source: Sci Rep. 2023 Aug 11;13:13055. doi: 10.1038/s41598-023-39772-5 (PMC10421893; doi:10.1038/s41598-023-39772-5)
Supplement: Supplementary file 1 — Supplementary Information 1. [file 41598_2023_39772_MOESM1_ESM.docx]

**Table S1** Investigated functional traits, their abbreviations, definitions, units of measurement, functional significance and respective literature reference, modified from Wellstein et al. (2013)

| Trait | Abbrevation | Description | Units | Functional Meaning | Literature |
| --- | --- | --- | --- | --- | --- |
| Leaf area | LA | One-sided area of an individual leaf | mm^2^ | Related to allometric factors, competitive ability and plant persistence | Pérez-Harguindeguy et al. (2013); Díaz et al. (2016) |
|  |  |  |  |  |  |
|  |  |  |  |  |  |
| Specific Leaf Area | SLA | Ratio of fresh leaf area to leaf dry mass | mm^2^/mg | Positively correlated with potential RGR and mass-based light-saturated photosynthetic rate, negatively with leaf longevity and C investment in secondary structural compounds. High values of SLA are associated with rapid production of biomass. Plasticity of SLA is well documented. | Kleyer et al. (2008); Perez-Harguindeguy et al. (2013); Wellstein et al. (2013); Westoby (1998) |
|  |  |  |  |  |  |
|  |  |  |  |  |  |
|  |  |  |  |  |  |
| Leaf Dry Matter Content | LDMC | Ratio of dry leaf mass to fresh leaf mass | mg/g | Positively correlated with leaf lifespan and structural investment, negatively with potential RGR. It scales with the inverse of SLA. Leaves with high values of LDMC tend to have a low turn-over enhancing nutrients conservation. | Kleyer et al. (2008); Perez-Harguindeguy et al. (2013); Wellstein et al. (2013) |
|  |  |  |  |  |  |
|  |  |  |  |  |  |
|  |  |  |  |  |  |
| Leaf Nitrogen Content | LNC | Total amount of of N per unit of dry leaf mass | mg/g | High LNC or LPC values are generally associated with high nutritional quality to the consumers in the food web. These traits vary significantly with environmental N and P available. | Perez-Harguindeguy et al. (2013) |
|  |  |  |  |  |  |
|  |  |  |  |  |  |
| Leaf Phosphorus Content | LPC | Total amount of of P per unit of dry leaf mass | mg/g |  | Perez-Harguindeguy et al. (2013) |
|  |  |  |  |  |  |
|  |  |  |  |  |  |

Table S1 continued

| Trait | Abbrevation | Description | Units | Functional Meaning | Literature |
| --- | --- | --- | --- | --- | --- |
| N:P ratio | N:P | Ratio between LNC and LPC | - | Used to assess the type of nutrient limitation. Optimal values of N:P ratio depend on species, growth rate, plant age and plant parts. Low N:P ratios indicate N limitation while some authors suggest that high N:P rations indicate P limitation alone. | Güsewell (2004); Güsewell and Koerselman (2002) |
| Stomatal Density | SD | Number of stomata/leaf surface | mm^-2^ | The two traits determine maximum leaf diffusive stomatal conductance of CO_2._  They reflect how stomatal behaviours respond to short-term environmental changes. | Franks and Beerling (2009); Wang et al. (2014) |
|  |  |  |  |  |  |
|  |  |  |  |  |  |
|  |  |  |  |  |  |
| Stomatal Length | SL | Length of stomata | µm |  |  |
|  |  |  |  |  |  |
|  |  |  |  |  |  |
|  |  |  |  |  |  |
|  |  |  |  |  |  |
|  |  |  |  |  |  |
|  |  |  |  |  |  |

**Fig. S1** Ice holes of (a) Cornacalda, (b) Appiano and (c) Lases. Photographs by Camilla Wellstein.


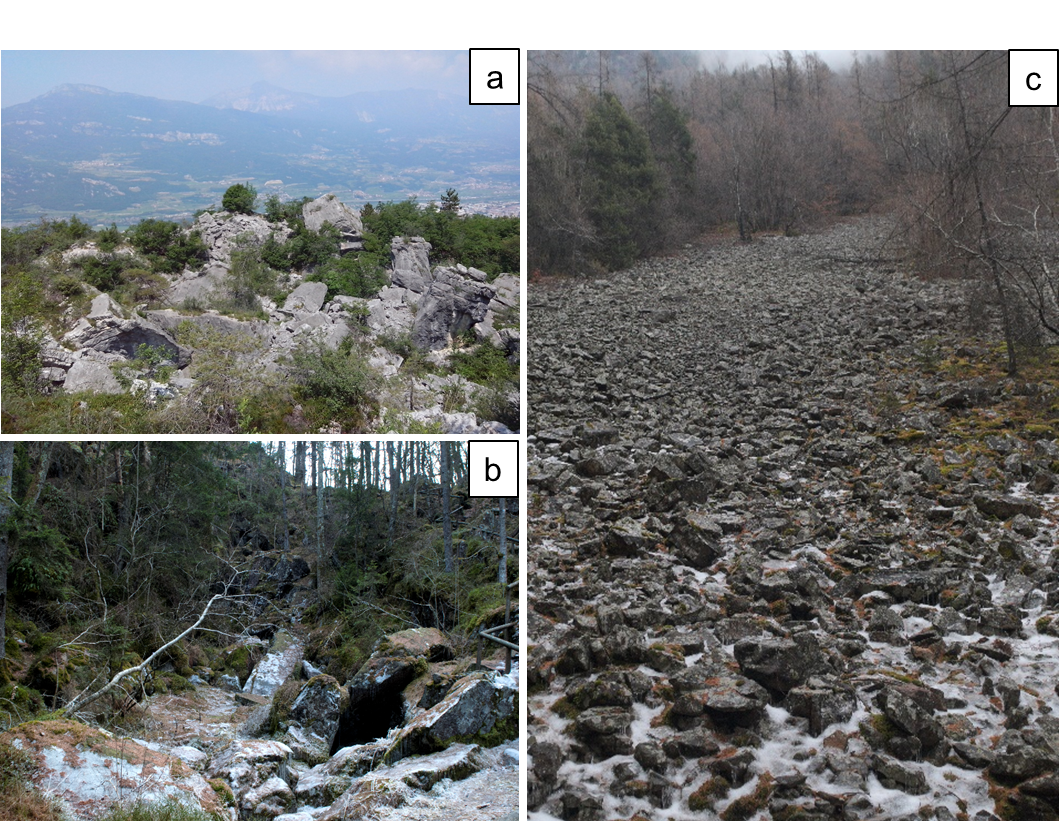


**Fig. S2** The clustered fineRADstructure coancestry heatmap of the individuals of *V. vitis-idaea*. The heatmap depicts variation in pairwise coancestry among individuals according to the scale shown on the right. The heatmap was created in R (R Development Core Team 2014, version 3.4.3) using the package fineRADstructure, a population inference package for RADseq data (available at [https://github.com/millanek/fineRADstructure](https://eur03.safelinks.protection.outlook.com/?url=https%3A%2F%2Fgithub.com%2Fmillanek%2FfineRADstructure&data=05%7C01%7CCamilla.Wellstein%40unibz.it%7C379c3fa3b7044f8f89d608db520ef575%7C9251326703e3401a80d4c58ed6674e3b%7C0%7C0%7C638193996442327577%7CUnknown%7CTWFpbGZsb3d8eyJWIjoiMC4wLjAwMDAiLCJQIjoiV2luMzIiLCJBTiI6Ik1haWwiLCJXVCI6Mn0%3D%7C3000%7C%7C%7C&sdata=T5MFMhPdttdzLErEIlV3HAlayunYJG2PXT8wliE1MKM%3D&reserved=0)) following this paper: Malinsky, M., Trucchi, E., Lawson, D. J., & Falush, D. RADpainter and fineRADstructure: Population Inference from RADseq Data. Molecular Biology and Evolution 35(5), 1284–1290 (2018).

**
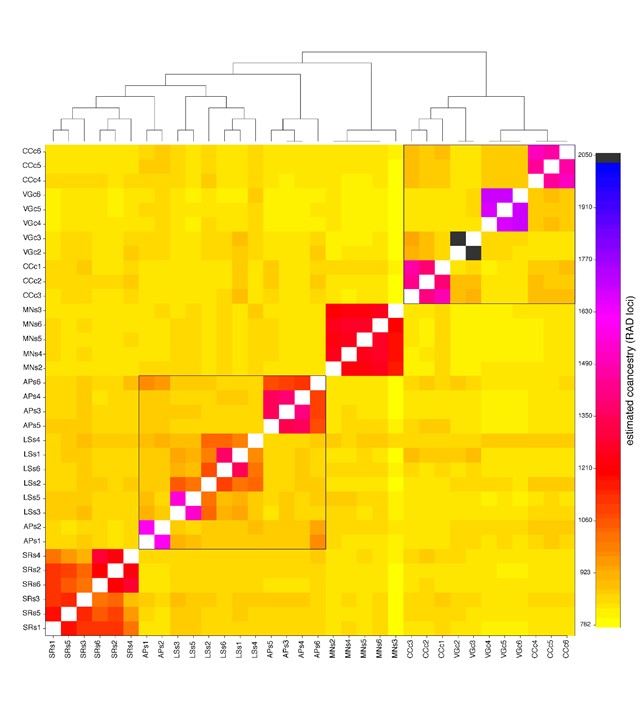
**
